# Lead Poisoning

**Published:** 1878-09

**Authors:** 


Several cases of lead poisoning which have lately occurred in Germany have been found to be due to the direct rays of the sun upon the oil cloth tops of baby carriages in which the children were kept when out of doors. The oil cloth is of American make, and contains forty-two per cent. of metallic lead.

